# Supplementary material for: Single-Cell RNA-Seq Revealed the Gene Expression Pattern during the In Vitro Maturation of Donkey Oocytes
Source: Genes (Basel). 2021 Oct 19;12(10):1640. doi: 10.3390/genes12101640 (PMC8535270; doi:10.3390/genes12101640)
Supplement: Supplementary file 1 [file genes-12-01640-s001.zip › genes-1413027-supplementary.pdf]

Table S1. Primers used for qRT–PCR.

Name	Sequence (5′-3′)	product length(bp)	Tm(°C)	Reference/accession numbers
GDF9-F	CCCCGCAAAGATACAACCCT	133	60	XM_014856804.1
GDF9-R	GGTCTTGGCACTGAGGAGTC			
BMP15-F	AGAGCCACTGTGGTTTACCG	187	60	XM_014827162.1
BMP15-R	GCCTTTGCCGAATGTGTTGT			
LGALS3-F	AAGGGAAGAAAGACAGGCGG	104	60	XM_014864690.1
LGALS3-R	GAGCATCATTGACCGCAACC			
ALG5-F	GCTGGTGAAGAATCGTGGGA	239	60	XM_014862690.1
ALG5-R	GTAGCGATGAAAACGTCCGC			
ACTB-R	CCACCATGTACCCAGGCATT	189	60	XM_014835097.1
ACTB-F	CGGACTCATCGTACTCCTGC			

Note: F, forward; R, reverse.
